# A novel two-layer SVM model in miRNA Drosha processing site detection

**DOI:** 10.1186/1752-0509-7-S4-S4

**Published:** 2013-10-23

**Authors:** Xingchi Hu, Chuang Ma, Yanhong Zhou

**Affiliations:** 1 Hubei Bioinformatics and Molecular Imaging Key Laboratory, College of Life Science and Technology, Huazhong University of Science and Technology, Wuhan, 430074, China; 2 School of Plant Sciences, University of Arizona, Tucson, AZ, USA

## Abstract

**Background:**

MicroRNAs (miRNAs) are a large class of non-coding RNAs with important functions wide spread in animals, plants and viruses. Studies showed that an RNase III family member called Drosha recognizes most miRNAs, initiates their processing and determines the mature miRNAs. The Drosha processing sites identification will shed some light on both miRNA identification and understanding the mechanism of Drosha processing.

**Methods:**

We developed a computational method for Drosha processing site predicting, named as DroshaPSP, which employs a two-layer mathematical model to integrate structure feature in the first layer and sequence features in the second layer. The performance of DroshaPSP was estimated by 5-fold cross-validation and measured by ACC (accuracy), Sn (sensitivity), Sp (specificity), P (precision) and MCC (Matthews correlation coefficient).

**Results:**

The results of testing DroshaPSP on the miRNA data of Drosophila melanogaster indicated that the Sn, Sp, and MCC thereof reach to 0.86, 0.99 and 0.86 respectively.

**Conclusions:**

We found the Shannon entropy, a chemical kinetics feature, is a significant feature in telling the true sites among the nearby sites and improving the performance.

## Background

MicroRNAs (miRNAs) are a large class of ~ 22nt long non-protein-coding RNAs that post-transcriptionally interfere the expression of their target genes by binding to the 3'-untranslated regions (3'UTR) [1]. MiRNAs were found to degrade or suppress the expression of great amount target genes [2,3] in plants, animals and viruses [4], which play important roles in embryo development, cell growth and tissue differentiation, apoptosis and proliferation, morphogenesis and so on [5-8].

Drosha is a Class 2RNase III enzyme. In most animals, except a few miRNAs which are produced by the miRtron pathway [9], it is Drosha that cleaves the long primary-miRNAs (pri-miRNAs) to precursor miRNA (pre-miRNA) hairpins of ~70nt in length [10], which initiates miRNA processing [11,12]. The Drosha processing step determines the sequence regions of pre-miRNAs for the sequentially biological process to produce mature miRNAs by Dicer. As Dicer selects cleavage sites by measuring a set distance from Drosha processing sites [13], Drosha is considered to be the key of making the determination of the mature miRNAs. Furthermore, the Drosha process also determines the efficiency and specificity of most miRNA expression [14]. Therefore, accurate identification of Drosha processing sites will facilitate the recognition of miRNAs and the mechanisms understanding of miRNA biogenesis.

The methods in both experimental and computational ways have been employed to identify the Drosha processing sites. Kadener et al. identified 137 Drosha target sites from pri-miRNAs at the genome scale of Drosophila experimentally with the tiling microarray technology [15]. Computational method is another option for quickly and low-costly identifying Drosha processing sites. The 'Microprocessor SVM' is a computational program used to identify human Drosha processing sites with the feature set formed by structure information features and base pair information features of pre-miRNA hairpin. However, the accuracy of 'Microprocessor SVM' predicting known 5'-Drosha processing sites in human is approximately 50% [16]. One of the possible reasons of the low accuracy may be the missing of some chemical kinetics features, such as the Shannon entropy of pre-miRNA folding.

In this study, we introduced a computational method named DroshaPSP that integrated the Shannon entropy [17] into the feature set to search Drosha processing sites on pre-miRNA hairpin structure. The Shannon entropy is verified to be an significant measure in non-coding RNA sequences (ncRNAs) folding, especially miRNA [18]. It is widely accepted that the pri-miRNA folding into hairpin structure is required for the Drosha processing, so we naturally infer that the Shannon entropy is important for Drosha processing step. As we expected, our Drosha processing site predicating program, called DroshaPSP, gave SN nearly 0.91 while SP was over 0.99, and the MCC reached 0.94. This result confirmed our hypothesis that chemical kinetics features, in particular, the Shannon entropy, are import for Drosha processing.

We have reported our research results to BIBM 2012 [19]. In this supplement, we are more specific on the Methods that how we established the two-layer classifier based on SVM and discuss the irreplaceability of the first layer.

## Methods

### Data

Drosophila melanogaster was chosen as the study species due to its small genome.

The Drosophila melanogaster miRNA annotation data, including the sequences of pre-miRNA, the structure data of miRNA hairpin, the sequences of mature miRNA and the sequences of miRNA star were downloaded from miRBase (http://www.mirbase.org/) [4], which collects the comprehensive annotation information of Drosophila melanogaster miRNAs. It should be noted that the miRNAs produced by miRtron pathway were not considered in this study, because they are not processed by Drosha.

The sequence data of Drosophila melanogaster genome were obtained from Ensemble database [20].

### Predicting steps of DroshaPSP

**Figure 1:**
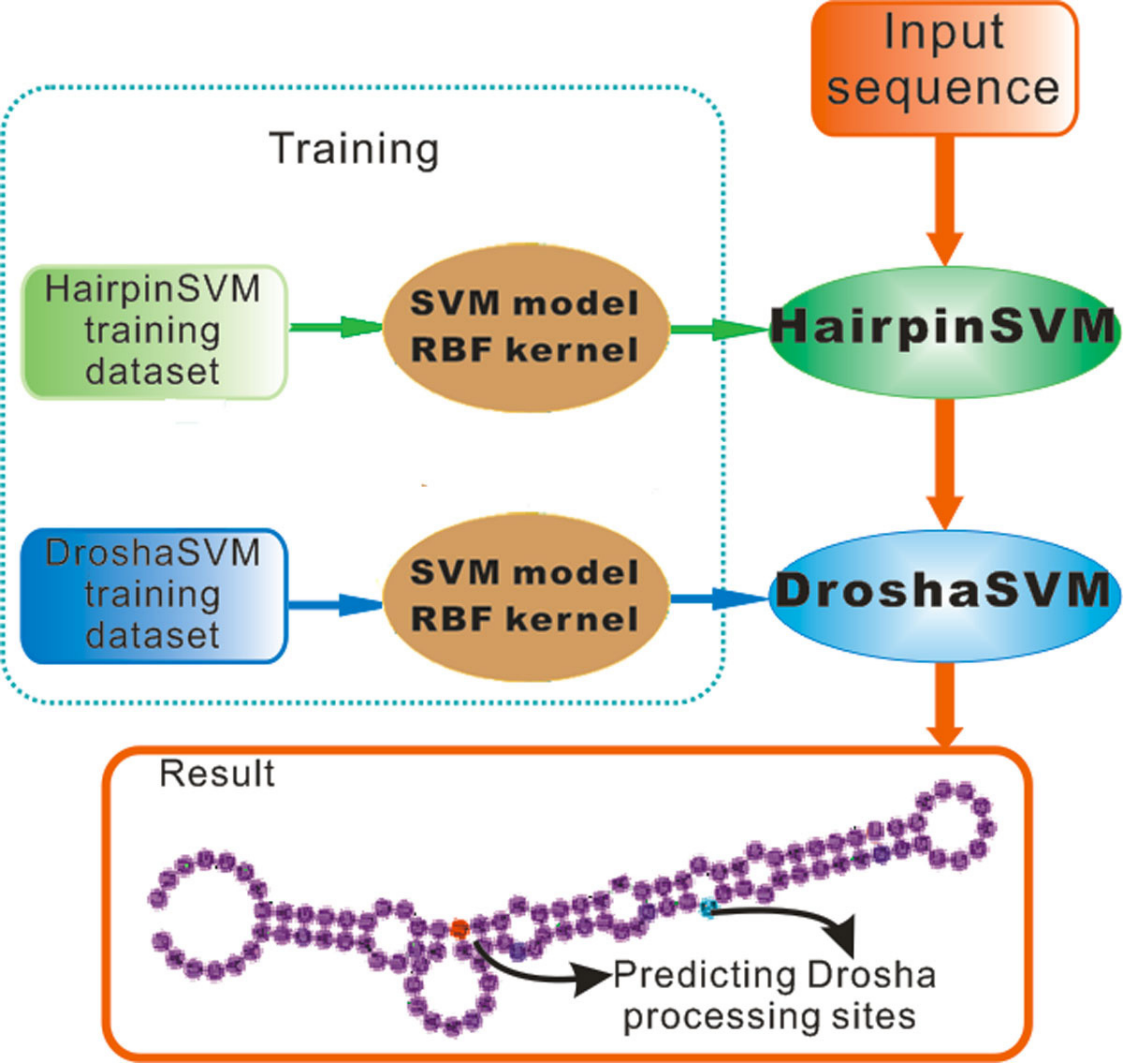
**The system architecture of DroshaPSP**. The DroshaPSP is composed of two SVM based classifiers, called HairpinSVM and DroshaSVM respectively. For a given input sequence, it is first folded and picked by HairpinSVM. If it is selected, the DroshaSVM is applied to predict Drosha processing sites.

A two-layer prediction model is used in DroshaPSP to predict the processing sites of Drosha, as shown in Figure 1. For a given gene sequence, DroshaPSP first determines the hairpin structure with the prediction model HairpinSVM, and then identifies the Drosha processing sites of the hairpin structure with the prediction model DroshaSVM, which integrates the structure, sequence and entropy information.

### HairpinSVM: Pre-miRNA like hairpin structure determination

HairpinSVM is a classifier that was constructed based on the support vector machine (SVM) [21] used for telling the pre-miRNA like hairpins which are the potential substrates of DroshaSVM. We selected the most widely used radial basis function kernel (RBF kernel) for HairpinSVM. The RBF kernel of SVM [22] was implemented with the package LIBSVM [23].

**Figure 2:**
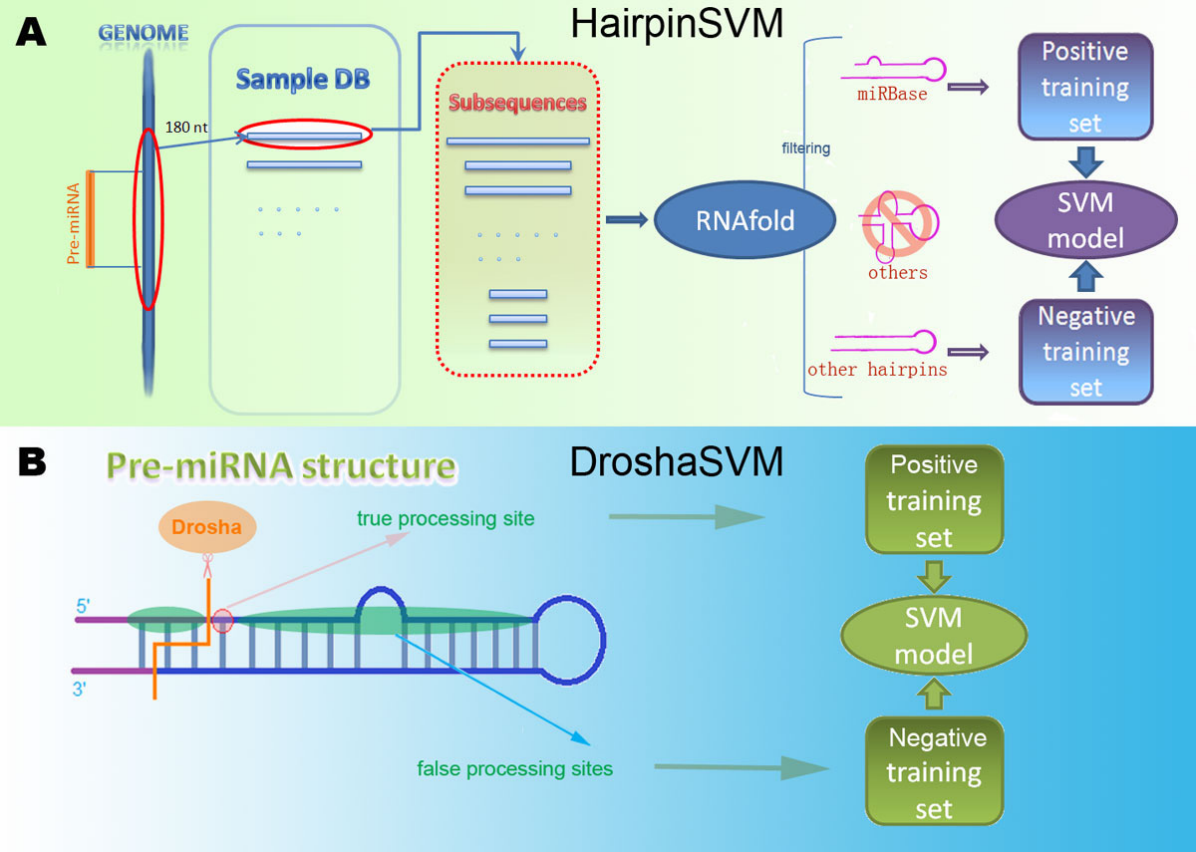
**HairpinSVM and DroshaSVM**. (A) The flow chart of HairpinSVM: for each pre-miRNA in miRBase, it is first mapped to the genome of Drosophila melanogaster and extended to 180nt. These 180nt sequences are collected into the Sample DB. For each sample in the Sample DB, all of its subsequences longer than 50nt are folded by RNAfold. After elimination of redundancy folding results, the ones same with the structure in miRBase are assembled in the true training set, other hairpin structures are assembled in false training set. (B) The flow chart of DroshaSVM: for each pre-miRNA hairpin structure, all the sites of 5' arm are accepted as Drosha processing site candidate, the true processing sites are based on miRBase annotation. Other sites compose false training set.

As shown in Figure 2A, HairpinSVM firstly mapped all the pre-miRNA sequences (70~100nt) obtained from miRBase to the Drosophila melanogaster genomic sequences by Blast [24], and extended to 180nt. These 180nt long sequences constituted the sample database (the Sample DB). For each sample in the Sample DB, all of its subsequences longer than 50nt are inputted to RNAfold software [25]. The hairpin structures returned by RNAfold were candidates for the HairpinSVM. In the case that the subsequences from a certain sample give out the same folding structure, only the longest one was retained. In brief, all the possible structures output by RNAfold were considered as pre-miRNA candidates. In the candidate dataset, the ones same with the corresponding pre-miRNA structure given out by miRBase formed the positive training set, others constituted negative training set. Finally we get 641 positive training samples and 3024 negative training samples for HairpinSVM.

In HairpinSVM, 12 structure features were included to tell the pre-miRNA like hairpin structures with the best possibility (Table 1).

**Table 1:** The features used in HairpinSVM

ID	Name	Description
**1**	Length	The length of the sequence
**2**	Loop_length	The loop size of hairpin structure
**3**	Stem_length	The stem length of hairpin structure
**4**	Pair	The number of base pairs in folding result
**5**	Pair_frac	The fraction of paired base in sequence
**7**	Insert_count	The number of bulges in the folding structure output by RNAfold
**6**	Insert_frac	The average length of bulges in sequence
**8**	Insert_count_frac	The ratio between the nucleotides in bulges and those in the sequence
**9**	Mfe	The minimal free energy output by RNAfold
**10**	Ensemble_fe	The free energy of the thermodynamic ensemble
**11**	Ensemble_fq	The probability of this single structure in the Boltzmann weighted ensemble of all structures.
**12**	Ensemble_div	The ensemble diversity is the average base-pair distance between all structures in the thermodynamic ensemble.

### DroshaSVM: Drosha processing site classifier

The output of DroshaSVM is the probability for each candidate of Drosha processing site. The candidates of Drosha processing sites refer to the sites at the 5'-stems of hairpins outputted by HairpinSVM (Figure 2B). Similar to Microprocessor SVM, we defined that the true Drosha processing sites are the 5'-ends of mature miRNAs and miRNA stars in 5'-stem of pre-miRNA hairpin annotated by miRBase. If miRBase gives no such annotation for a pre-miRNA hairpin, we presumed that 3'-ends of mature miRNAs gave a 2nt overhang to relative 5'-true Drosha processing site. For DroshaSVM training, we collected 641 positive samples with experimentally validated from miRBase database. The negative sample set is formed by other 30,873 sites in 5'-stems of known pre-miRNAs.

Like the HairpinSVM, DroshaSVM also adopt RBF kernel for prediction model. Besides the normally used features, such as the base pair and its probability, the length from the loop, we also integrated the entropy features into DroshaSVM (Table 2). The Shannon entropy is a Dynamical feature, which has been verified to be an significant measure in non-coding RNA sequences (ncRNAs) folding, especially miRNA. The scaled values of the features were input to SVM model training.

**Table 2:** The features used in DroshaSVM

ID	Name	Description
**1**	Loop_Distance	Distance from processing site candidate to loop of the hairpin structure.
**2~11**	Structure	Structure description of the candidate site and 9nt sites forward are paired or not.
**12~21**	Base	The base types of the candidate site and 9nt sites forward.
**22~31**	Probability	The base pairing probability of the candidate site and 9nt sites forward.
**32~41**	Entropy	The Shannon entropy of the candidate site and 9nt sites forward.

### Estimating the performance

We applied 5-fold cross-validation test on both prediction models. In brief, both the positive and negative samples are firstly divided into 5 folds randomly. The classifier is then trained with data from 4 folds and tested on data from the rest one fold in turn. According to the results of 5-fold cross-validation, five widely used measures are used to estimate the performance of both HairpinSVM and DroshaSVM, which are: ACC (accuracy), Sn (sensitivity), Sp (specificity), P (precision) and MCC (Matthews correlation coefficient). The measures are defined as follow:

$$\text{ACC}=\frac{\text{TP}+\text{TN}}{\text{TP}+\text{FP}+\text{TN}+\text{FN}}$$

$$\text{SN}=\frac{\text{TP}}{\text{TP}+\text{FN}}$$

$$\text{SP}=\frac{\text{TN}}{\text{TN}+\text{FP}}$$

$$\text{P}=\frac{\text{TP}}{\text{TP}+\text{FP}}$$

$$\text{MCC}=\frac{\text{TP}\times \text{TN}-\text{FN}\times \text{FP}}{\sqrt{\left(\text{TP}+\text{FN}\right)\left(\text{TN}+\text{FP}\right)\left(\text{TP}+\text{FP}\right)\left(\text{FN}+\text{FN}\right)}}$$

where TN, TP, FN and FP respectively represent the counts of true negative, true positive, false negative, false positive. Unusually, the MCC, instead of the ACC, is used to estimate the overall performance and determine the default threshold due to the unbalanced positive and negative training sets [26].

To estimate the classifiers comprehensively, the receiver operating characteristic curve (ROC curve) is used to present the performance intuitively.

The DroshaPSP program was tested by the testing dataset and the performance is accessed also by ACC, SN, SP, P and MCC.

## Results

We developed a program called DroshaPSP to automatically identify the Drosha processing sites from the given sequence based on SVM method. For a given sequence, it was first told by HairpinSVM if it is a pre-miRNA-like hairpin structure. If it's predicted as a positive sample by HairpinSVM, then the DroshaSVM determined whether there were Drosha processing sites and where they would be.

### Performance of the classifiers

**Figure 3:**
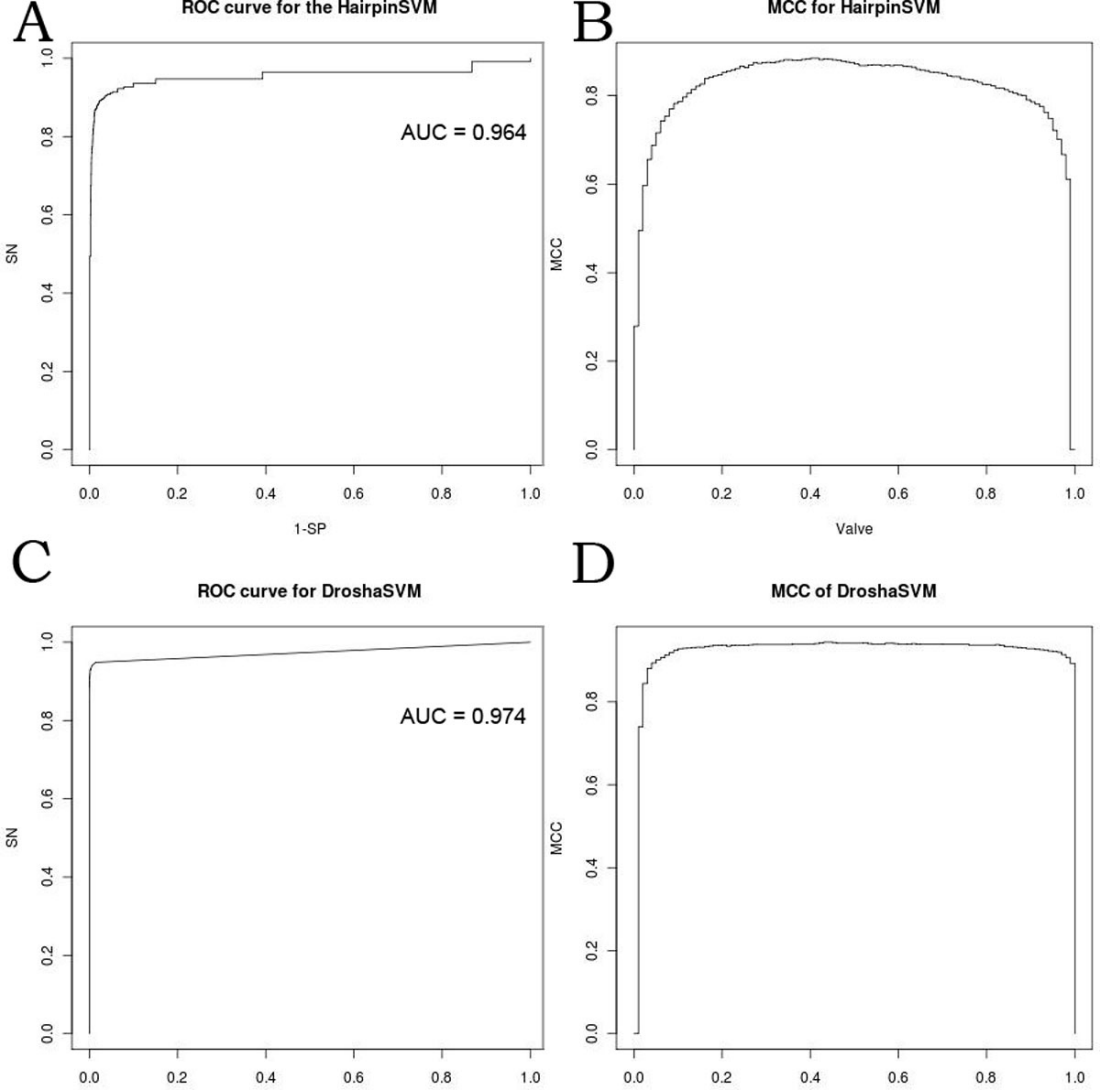
**The performance of HairpinSVM and DroshaSVM**. (A) The ROC curve for HairpinSVM with the AUC = 0.964. (B) The MCC with the valve curve of HairpinSVM. (C) The ROC curve for DroshaSVM with the AUC = 0.974. (D) The MCC with the valve curve of DroshaSVM.

We used radial basis function kernel for both the HairpinSVM and DroshaSVM, then tested them by 5-fold cross validation. The HairpinSVM was trained by the training dataset with 641 positive samples and 3024 negative samples. The HairpinSVM gave out excellent performance with the parameters nu = 0.121 and gamma = 64, the MCC reached to 0.882, while SN was 0.867, SP increased to 0.988, the ACC and P were 0.967 and 0.938. The ROC curve is shown in Figure 3A. The AUC of ROC curve for the HairpinSVM is 0.964. For the DroshaSVM, the size of true training set and false training set were 641 and 30873. The DroshaSVM gave out the performance that with SN = 0.908, SP = 0.999, the MCC reached 0.944 and the ACC was 0.998, the value of P was 0.983, MCC 0.944. The ROC curve of DroshaSVM performance is shown in Figure 3C. The AUC under the ROC curve represent the performance of DroshaSVM is 0.974. Because of the unbalanced training dataset, the MCC value to different threshold of HairpinSVM and DroshaSVM are shown in Figure 3B and Figure 3D, which indicate that the performance of HairpinSVM and DroshaSVM were stable. The test results suggested that the HairpinSVM and DroshaSVM gave the reliable results of pre-miRNA hairpin structure and Drosha processing sites prediction.

### Performance of the DroshaPSP program

For the whole prediction program testing, we used all miRNAs of Drosophila melanogaster in miRBase version 18.0 as the testing set. The test showed that SN was 0.859 while SP reached 0.999, the value of ACC and P were 0.998 and 0.870. The comprehensive measurement MCC achieved 0.864.

### Estimating the importance of the features

It is meaningful for us to estimate the influence of each feature to the SVM classifiers, so that we could figure out that the importance of each feature and get a better understanding of the miRNA maturation. To this aim, the F-score method is applied. F-score is an effective method to estimate the discrimination of two sets. Given training vectors x_k_, k = 1, ..., m, the number of positive and negative instances are marked as n+ and n-, respectively, then for the *i*th feature, its F-score is calculated as:

$$\text{F}\left(i\right)\frac{\left({\bar{x_{i}}}^{\left(+\right)}-\bar{x_{i}}\right)+{\left({\bar{x_{i}}}^{\left(-\right)}-\bar{x_{i}}\right)}^{2}}{\frac{1}{n_{+}-1}\sum_{k=1}^{n_{+}}{\left(x_{k,i}^{\left(+\right)}-{\bar{x}}_{i}^{\left(+\right)}\right)}^{2}+\frac{1}{n_{-}-1}\sum_{k=1}^{n_{-}}{\left(x_{kj}^{\left(-\right)}-{\bar{x_{i}}}^{\left(-\right)}\right)}^{2}}$$

where $\bar{{\text{x}}_{\text{i}}}$, ${\bar{{\text{x}}_{\text{i}}}}^{\left(+\right)}$, ${\bar{{\text{x}}_{\text{i}}}}^{\left(-\right)}$ are the average of the *i*th feature of the whole, positive, and negative data sets, respectively; ${\text{x}}_{\text{k},\text{i}}^{\left(+\right)}$and ${\text{x}}_{\text{k},\text{i}}^{\left(-\right)}$ are the *i*th feature of the *k*th positive and negative instance. The larger the F-score is, the more likely this feature is discriminative.

**Figure 4:**
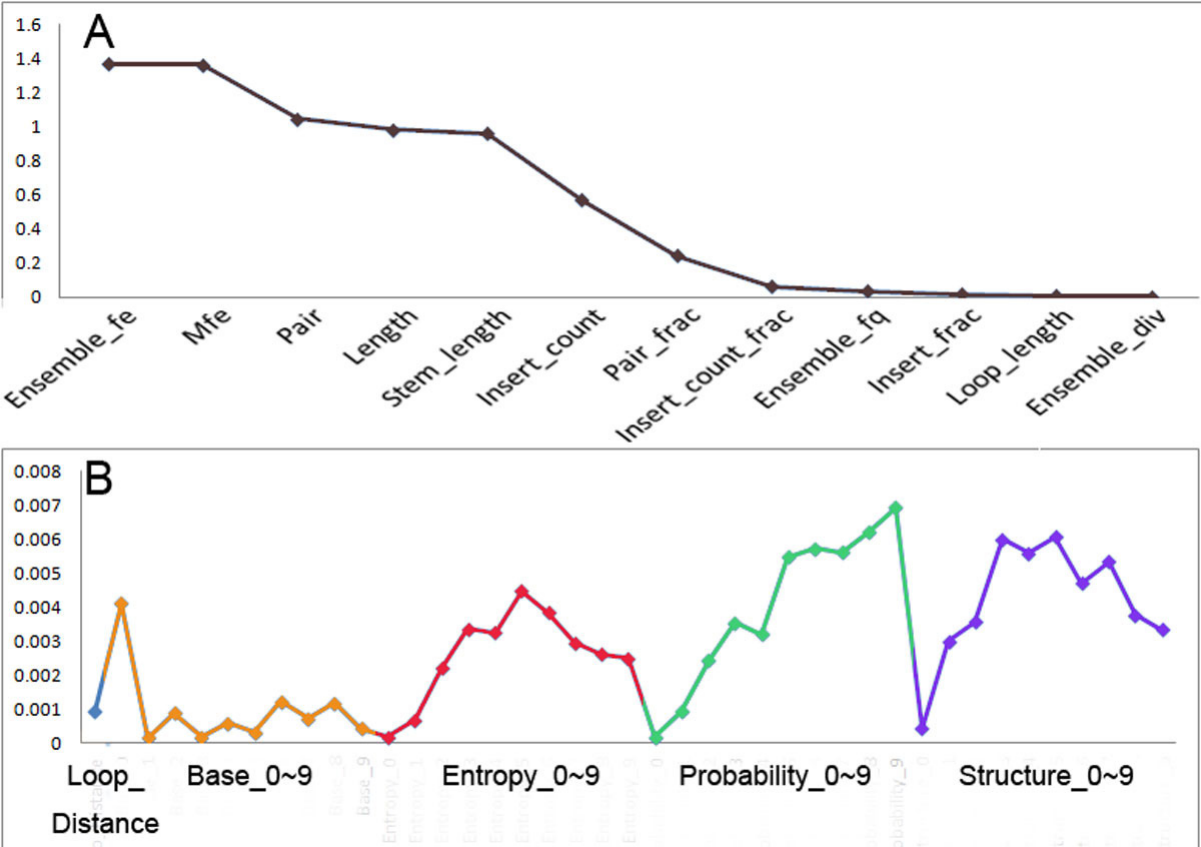
**The F-score of feature set of HairpinSVM and DroshaSVM**. (A) The F-score for HairpinSVM, with F-score descending. (B) The F-score for DroshaSVM, different feature classes are marked different colors.

The Figure 4A and Figure 4B present the F-score of each feature used in HairpinSVM and DroshaSVM respectively. The F-score of the feature stands for its contribution to the classifier. We can see in Figure 4A that the energy features, including the free energy of the thermodynamic ensemble and the minimal free energy, are the most effective features for pre-miRNA like hairpin selection. The features of stem structure took the second place, such as pair, length, and stem length. Other structure features of stem which impact the balance of the 5' stem and 3' stem, such as the number of bulges in the folding structure and the fraction of paired base in sequence, only contributed a little to HairpinSVM. According to Figure 4A, the loop structural features are less important than those features about stem. For DroshaSVM, the F-scores of all the used features are as showed in Figure 4B. Unexpectedly, the F-score of the base types is low in all the sites we selected. These facts suggest that the base types are not so important, and the stability and probability of the base pairs of these sites are effective features for Drosha processing site prediction. We found that the region from position 3 to position 9 has higher F-score, which may be the functional positions in Drosha process. However, different features have specific high F-score regions. The entropy got highest F-score in position 5 and 6, the base pairing probability and structure got relatively higher scores, especially the probability of position 8 and 9. In addition, all the features of candidate sites got low F-scores. The explanation for this observation may be that the processing sites themselves have little to do with the Drosha processing site determination.

### The Shannon entropy affects the Drosha process

As far as we know that the Shannon entropy is used in the Drosha processing site identification for the first time. The Shannon entropy is a powerful chemical kinetics feature which has been proved to be effective in ncRNA folding [18]. According to the F-score analysis result (Figure 4), the traditional features probability and structure information got high F-score, the Shannon entropy showed effect that should not be ignored. The F-score of the Shannon entropy were higher than the information of base pair in candidate site and sites forward. Once we removed the Shannon entropy, the modified feature set gave out the performance that the AUC under the ROC curve of DroshaSVM decreased 9% (AUC = 0.886).

**Figure 5:**
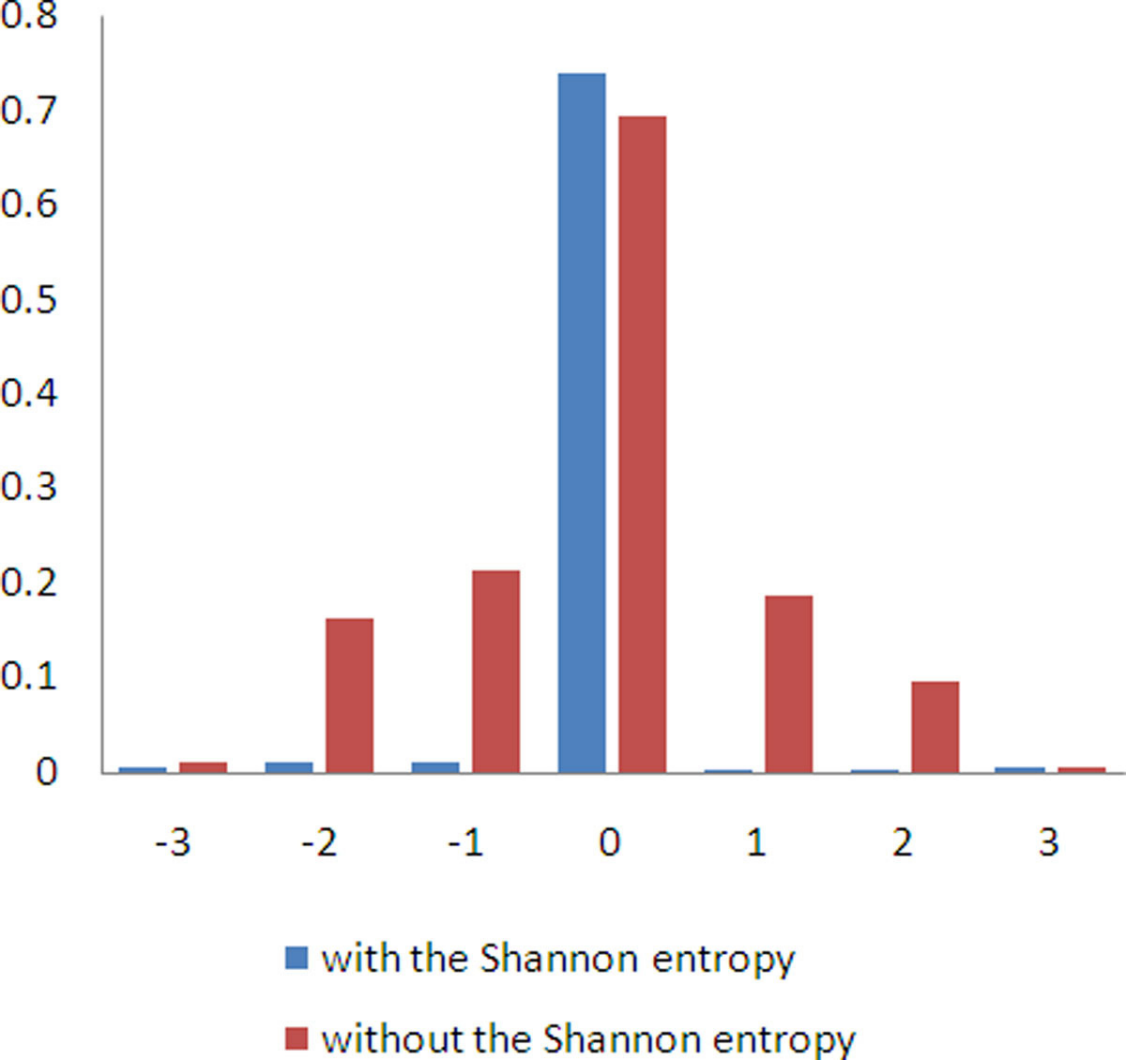
**The average DroshaSVM output score with and without the Shannon entropy**. The blue and red histograms present the average scores of true Drosha processing sites and 3nt upstream and downstream sites given by DroshaSVM using the feature sets included Shannon entropy and not.

We did a survey on he scores calculated by DroshaSVM with the feature set included or removed the Shannon entropy in the region of 3nt downstream and upstream to the true Drosha processing sites. The Figure 5 is the histogram that shows the average score calculated by DroshaSVM of the sites with different distance to true Drosha processing sites in both cases. The figure clearly shows that the average score of true Drosha processing sites is much higher than the sites nearby while applying the feature set included the Shannon entropy, and there is no significant difference between the sites with different distance from the true Drosha processing sites. If the feature set without the Shannon entropy is used, the average score of neighboring sites within 2nt showed a remarkable increase depending on distance from true processing sites.

These experiments demonstrated that the feature Shannon Entropy is a significant feature to tell Drosha processing sites and indicated that the Drosha process is influenced by the chemical kinetics of pre-miRNA folding.

## Discussion and conclusion

The precise detection of Drosha processing sites is a crucial procedure for miRNA identification and the revealing of miRNA maturation. In this study, we proposed a two-layer prediction model named DroshaPSP to identify Drosha processing sites by combining the sequence and structure information, and the evaluation results show that our method can achieve high prediction accuracy.

In our model, a novel dynamical feature was introduced, Shannon entropy, which is helpful to distinguish the true processing sites from the ones that nearby. In the previous study, the true processing sites and the neighboring sites within 2nt are indistinct due to the similar scores assigned by their Microprocessor SVM, which led to a serious problem in predicting Drosha processing site. Finding the features that can sufficiently characterize the genuine Drosha processing sites from the neighboring ones is our prime interest. Of this purpose, we brought in the Shannon entropy, which is a novel dynamical feature. As showed in Figure 5, with the Shannon entropy, DroshaPSP can pinpoint the true processing site from the neighborhood clearly.

Drosophila melanogaster was chosen as our study species, due to its extended annotation of Drosha processing sites on miRNAs. We did not compare our DroshaPSP with Microprocessor SVM, because the parameters of latter method were derived from human miRNAs, which were reported to be quite different from Drosophila melanogaster miRNAs, such as different cleavage partners of Drosha in human and Drosophila. Thus, the direct comparison of two prediction models derived from these two distinct species would bring on unfair results.

It is noteworthy that the purpose of HairpinSVM, the first layer of DroshaPSP, is not to scan the pre-miRNA from the given sequence, but to select the pre-miRNA like hairpin structure from all the RNA folding results of the given sequence. So, HairpinSVM cannot be replaced by other pre-miRNA predicting program. In order to clearly classify the pre-miRNA like hairpin structures, negative samples should be carefully chosen. Our negative samples are close with the positive samples in location and sequence but with clearly different hairpin structure, which make our negative samples very suitable and lead to a good performance of the first layer classification.

Although our proposed two-layer SVM method has high prediction accuracy, it is rather time-consuming, due to a lot of folding work done by RNAfold which is highly computational demanding. For example, predicting a 180nt sequence requires more than 3 minutes. This shortcoming limited its application in large dataset.

In the future, we will try to cut down the run time by changing programming language and improve the prediction accuracy of DroshaPSP with more structure features including the structure, base probability, entropy for each site. We will also extensively evaluate the performance of DroshaPSP with the prediction model trained from Drosha processing sites from other species. In addition, we are planning to develop a stand-alone implement with parallel computation option for Drosha processing site recognition on different OS platforms.

In conclusion, we developed a Drosha processing site predicting program, called DroshaPSP, which is composed of two classifiers based on SVM, the HairpinSVM and the DroshaSVM. The HairpinSVM gave out the performance with MCC 0.88, and the DroshaSVM was even better with the MCC reaching 0.94. The overall performance of DroshaSVM was that MCC reached 0.86 while SN was equal to 0.86 and SP was over 0.99. We brought the Shannon Entropy in the feature set of DroshaPSP for the first time, and gained a substantial improvement. It is found that the Shannon Entropy helped the DroshaSVM in telling the true processing site from the neighborhood.

## Competing interests

The authors declare that they have no competing interests.

## Authors' contributions

All authors designed the experiment. XH carried out data collection and reduction, trained the prediction model and drafted the manuscript. CM drafted the manuscript and revised it. YZ provided the idea and approval the final version.

## Declarations

The publication costs for this article were funded by the Ministry of Education of China (20050487037), the Program for New Century Excellent Talents in University (NCET-060651), the National Platform Project of China (2005DKA64001), the National Natural Science Foundation of China (90608020 and 30971642), and Natural Science Foundation of Hubei Province of China (2009CDA161).

This article has been published as part of *BMC Systems Biology *Volume 7 Supplement 4, 2013: Selected articles from the IEEE International Conference on Bioinformatics and Biomedicine 2012: Systems Biology. The full contents of the supplement are available online at http://www.biomedcentral.com/bmcsystbiol/supplements/7/S4.

## References

[B1] Bartel DP MicroRNAs: Genomics, biogenesis, mechanism, and function (Reprinted from Cell, vol 116, pg 281-297, 2004) Cell 2007 131 4 11 29 10.1016/s0092-8674(04)00045-5 14744438

[B2] Lim LP Lau NC Garrett-Engele P Grimson A Schelter JM Castle J Bartel DP Linsley PS Johnson JM Microarray analysis shows that some microRNAs downregulate large numbers of target mRNAs Nature 2005 433 7027 769 773 10.1038/nature03315 15685193

[B3] Vasudevan S Tong Y Steitz JA Switching from repression to activation: microRNAs can up-regulate translation Science 2007 318 5858 1931 1934 10.1126/science.1149460 18048652

[B4] Griffiths-Jones S Saini HK van Dongen S Enright AJ miRBase: tools for microRNA genomics Nucleic Acids Research 2008 36 D154 D158 10.1093/nar/gkn221 17991681 PMC2238936

[B5] Bartel DP MicroRNAs: genomics, biogenesis, mechanism, and function Cell 2004 116 2 281 297 10.1016/S0092-8674(04)00045-5 14744438

[B6] Cheng AM Byrom MW Shelton J Ford LP Antisense inhibition of human miRNAs and indications for an involvement of miRNA in cell growth and apoptosis Nucleic Acids Res 2005 33 4 1290 1297 10.1093/nar/gki200 15741182 PMC552951

[B7] Harfe BD MicroRNAs in vertebrate development Curr Opin Genet Dev 2005 15 4 410 415 10.1016/j.gde.2005.06.012 15979303

[B8] Wienholds E Kloosterman WP Miska E Alvarez-Saavedra E Berezikov E de Bruijn E Horvitz HR Kauppinen S Plasterk RH MicroRNA expression in zebrafish embryonic development Science 2005 309 5732 310 311 10.1126/science.1114519 15919954

[B9] Okamura K Hagen JW Duan H Tyler DM Lai EC The mirtron pathway generates microRNA-class regulatory RNAs in Drosophila Cell 2007 130 1 89 100 10.1016/j.cell.2007.06.028 17599402 PMC2729315

[B10] Han JJ Lee Y Yeom KH Kim YK Jin H Kim VN The Drosha-DGCR8 complex in primary microRNA processing Genes & Development 2004 18 24 3016 3027 10.1101/gad.1262504 15574589 PMC535913

[B11] Lee Y Ahn C Han J Choi H Kim J Yim J Lee J Provost P Radmark O Kim S et al The nuclear RNase III Drosha initiates microRNA processing Nature 2003 425 6956 415 419 10.1038/nature01957 14508493

[B12] Vermeulen A Behlen L Reynolds A Wolfson A Marshall WS Karpilow J Khvorova A The contributions of dsRNA structure to Dicer specificity and efficiency Rna-a Publication of the Rna Society 2005 11 5 674 682 10.1261/rna.7272305 PMC1370754 15811921

[B13] Park J-E Heo I Tian Y Simanshu DK Chang H Jee D Patel DJ Kim VN Dicer recognizes the 5[prime] end of RNA for efficient and accurate processing Nature 2011 475 7355 201 205 10.1038/nature10198 21753850 PMC4693635

[B14] Feng Y Zhang X Song Q Li T Zeng Y Drosha processing controls the specificity and efficiency of global microRNA expression Biochim Biophys Acta 2011 1809 11-12 700 707 10.1016/j.bbagrm.2011.05.015 21683814 PMC3210421

[B15] Kadener S Rodriguez J Abruzzi KC Khodor YL Sugino K Marr MT Nelson S Rosbash M Genome-wide identification of targets of the drosha-pasha/DGCR8 complex RNA 2009 15 4 537 545 10.1261/rna.1319309 19223442 PMC2661833

[B16] Helvik SA Snove O Saetrom P Reliable prediction of Drosha processing sites improves microRNA gene prediction Bioinformatics 2007 23 2 142 149 10.1093/bioinformatics/btl570 17105718

[B17] Huynen M Gutell R Konings D Assessing the reliability of RNA folding using statistical mechanics J Mol Biol 1997 267 5 1104 1112 10.1006/jmbi.1997.0889 9150399

[B18] Freyhult E Gardner PP Moulton V A comparison of RNA folding measures Bmc Bioinformatics 2005 6 241 10.1186/1471-2105-6-241 16202126 PMC1274297

[B19] Hu X Zhou Y Ma C Recognizing drosha processing sites by a two-step prediction model with structure and sequence information Bioinformatics and Biomedicine (BIBM), 2012 IEEE International Conference on: 4-7 October 2012 2012 1 4 10.1109/BIBM.2012.6392714

[B20] Hubbard T Barker D Birney E Cameron G Chen Y Clark L Cox T Cuff J Curwen V Down T The Ensembl genome database project Nucleic Acids Research 2002 30 1 38 41 10.1093/nar/30.1.38 11752248 PMC99161

[B21] Boser BE Guyon IM Vapnik VN A training algorithm for optimal margin classifiers 1992: ACM 1992 144 152

[B22] Burges CJC A tutorial on Support Vector Machines for pattern recognition Data Min Knowl Discov 1998 2 2 121 167 10.1023/A:1009715923555

[B23] Chang CC Lin CJ LIBSVM: a library for support vector machines ACM Transactions on Intelligent Systems and Technology (TIST) 2011 2 3 27

[B24] McGinnis S Madden TL BLAST: at the core of a powerful and diverse set of sequence analysis tools Nucleic Acids Research 2004 32 W20 W25 10.1093/nar/gkh435 15215342 PMC441573

[B25] Hofacker IL Fontana W Stadler PF Bonhoeffer LS Tacker M Schuster P Fast folding and comparison of RNA secondary structures Monatshefte für Chemie/Chemical Monthly 1994 125 2 167 188 10.1007/BF00818163

[B26] Baldi P Brunak S Chauvin Y Andersen CAF Nielsen H Assessing the accuracy of prediction algorithms for classification: an overview Bioinformatics 2000 16 5 412 424 10.1093/bioinformatics/16.5.412 10871264

